# Expression of mRNAs for pro-and anti-apoptotic factors in granulosa cells and follicular fluid of women undergoing in vitro fertilization. A pilot study

**DOI:** 10.1186/s12884-021-03834-1

**Published:** 2021-05-24

**Authors:** József Bódis, Endre Sulyok, Ákos Várnagy, Viktória Prémusz, Krisztina Gödöny, Alexandra Makai, Ágnes Szenczi, Timea Varjas

**Affiliations:** 1 ELKH-PTE Human Reproduction Scientific Research Group, University of Pécs, Édesanyák u. 17., Pécs, H-7624 Hungary; 2 Doctoral School of Health Sciences, Faculty of Health Sciences, University of Pécs, Vörösmarty u. 4., Pécs, H-7621 Hungary; 3 Department of Obstetrics and Gynaecology, Medical School, University of Pécs, Édesanyák u. 17., Pécs, H-7624 Hungary; 4 Department of Public Health Medicine, Medical School, University of Pécs, Szigeti u. 12., Pécs, H-7621 Hungary

**Keywords:** Apoptosis, Follicular fluid, Granulosa cell, In vitro fertilization, mRNA expression

## Abstract

**Background:**

This observational clinical study evaluated the expression levels and predictive values of some apoptosis-related genes in granulosa cells (GCs) and follicular fluid (FF) of women undergoing in vitro fertilization (IVF).

**Methods:**

GCs and FF were obtained at oocyte retrieval from 31 consecutive patients with heterogeneous infertility diagnosis (age: 34.3 ± 5.8 years, body mass index: 24.02 ± 3.12 kg/m2, duration of infertility: 4.2 ± 2.1 years). mRNA expression of pro-apoptotic (BAX, CASP3, CASP8) and anti-apoptotic (BCL2, AMH, AMHR, FSHR, LHR, CYP19A1) factors was determined by quantitative RT-PCR using ROCHE LightCycler 480.

**Results:**

No significant difference in GC or FF mRNA expression of pro- and anti-apoptotic factors could be demonstrated between IVF patients with (9 patients) or without (22 patients) clinical pregnancy. Each transcript investigated was detected in FF, but their levels were markedly reduced and independent of those in GCs. The number of retrieved oocytes was positively associated with GC AMHR (*r* = 0.393, *p* = 0.029), but the day of embryo transfer was negatively associated with GC LHR (*r* = − 0.414, *p* = 0.020) and GC FSHR transcripts (*r* = − 0.535, *p* = 0.002). When pregnancy positive group was analysed separately the impact of apoptosis- related gene expressions on some selected measures of IVF success could be observed. Strong positive relationship was found between gene expression levels of pro- and anti-apoptotic factors in GCs.

**Conclusion:**

Our study provides only marginal evidences for the apoptosis dependence of IVF outcome and suggests that the apoptosis process induces adaptive increases of the anti-apoptotic gene expression to attenuate apoptosis and to protect cell survival.

## Background

Convincing evidence have been provided for the prominent role of apoptosis in female reproduction [1–3]. Apoptosis of ovarian cells is intimately involved in the regulation of folliculogenesis, gametogenesis, fertilization and embryonic development. It is a physiological process provided that the pro-apoptotic factors are in balance with factors promoting cell survival. When pro-apoptotic factors predominate over anti-apoptotic ones the finely tuned complex interaction between the two opposing systems is disturbed, apoptosis accelerates, the ovarium reserve declines, and early ovarium aging occurs. As a result, the female fertilization potential is compromised [4–10]. Based on these observations the apoptosis-related elements have been widely investigated and they have been claimed to be reliable markers to predict in vitro fertilization (IVF) outcome [11].

These observations have revealed that in mammalian ovaries the apoptosis of granulosa cells (GCs) and oocytes has deleterious effects on oocyte maturation, fertilization and embryonic development in vitro and in vivo. Apoptotic cell death is characterized morphologically by cytoplasmic and chromatin condensation, membrane blebbing, fragmentation of cells into membrane-bound apoptotic bodies and phagocytosis by the neighbouring cells. Biochemically two major death pathways have been identified that are involved in the initiation of ovarian apoptosis: a/ the transmembrane death receptor Fas and its ligand- FasL system when activated induces the release of pro-apoptotic proteins (procaspase-8 and procaspase-3) [12] and b/ mitochondrial and endoplasmatic reticulum-associated pathways. In the former cytochrome c release interferes with electron transport, cellular redox status and liberates caspase (CASP) activator proteins [13]. In the latter endoplasmic reticulum stress induces apoptosis via the function of CASP12 [14].

The current view that apoptosis of GCs can be used to characterize the competence of oocytes and pre-implantation embryos, however, has been recently challenged [8, 15]. In a most recent review by Regan et al. it is written “apoptosis levels of the GCs are reflective of the proliferative stage of the follicle rather than a predictor of oocyte health”. Accordingly, its predictive value for oocyte quality and ensuring pregnancy rate is poor [8].

With respect to these apparent controversies the present study was designed to measure the expression profile of apoptosis-related genes in patients undergoing IVF. Specifically, we determined the mRNA expression of pro-apoptotic factors CASP3, CASP8 and BCL2 associated X apoptosis regulator (BAX), as well as that of anti-apoptotic factors BCL2 apoptosis regulator, FSH receptor (FSHR), LH receptor (LHR), anti-Müllerian hormone (AMH), anti-Müllerian hormone receptor type 2 (AMHR 2) and cytochrome P450 family 19 subfamily A member 1 (CYP19A1) in GCs and cell-free follicular fluid (FF) obtained during oocyte retrieval. Attempt was also made to relate these genes to the outcome measures of IVF (number of mature oocytes, viable embryos and pregnancy rate).

## Material and methods

### Patients

This observational, clinical study was carried out between 1 September 2019 and 1 December 2019 in the Assisted Reproduction Unit, Department of Obstetrics and Gynecology, University of Pécs, Pécs, Hungary. The study comprised 31 consecutive patients who were indicated for fertility treatment. Eligible patients were recruited according to the data of fertility consultation. They did not have metabolic or vascular diseases (obesity, diabetes mellitus, metabolic syndrome, fatty liver diseases and atherosclerosis) or renal diseases. Enrolment of patients into the IVF procedure was approved by two independent physicians. Superovulation treatment, fertilization methods and embryo selection were performed according to standard protocols as described in our previous publication [16]. As controlled ovarium stimulation has been shown to influence apoptosis [17, 18] details of our standard procedures are given as follows: Inducing IVF GnRH agonist triptorelin (Gonapeptyl, Ferring, Germany) was used in a long or short protocol, and the stimulation was performed with individual dosage of rFSH (Gonal-F; Serono Aubonne, Switzerland) varying from 100 to 225 IU per day depending on the follicular maturation. The starting dose was adopted according to the BMI and the age. For patients with a previously known low response it was increased to a maximum dose of 300–350 IU daily. No discernible differences could be detected in the cumulative dosage of GnRH or rFSH between women with or without chemical/clinical pregnancy. The major clinical and laboratory characteristics of the patients are summarized in Table 1.

**Table 1:** The major clinical and laboratory characteristics of IVF patients who progressed to chemical/clinical pregnancy and those who failed to become pregnant. (mean ± SD)

	All patients	Pregnancy- negative group	Pregnancy- positive group	Intergroup difference
	(*n* = 31)	(*n* = 20)	(*n* = 11)	*p*
Age (years)	34.35 ± 5.84	35.45 ± 6.21	31.67 ± 3.91	0.030
BMI (kg/m^2^)	24.02 ± 3.12	24.06 ± 3.29	23.92 ± 2.85	0.273
Female infertility, n (%)	14 (45.16)	9 (45.00)	5 (45.45)	0.981
Male infertility, n (%)	17 (54.84)	11 (55.00)	6 (54.55)	
Number of previous IVF, n	2.10 ± 1.45	2.18 ± 1.53	1.89 ± 1.27	0.660
Serum oestradiol (pmol/l)	2919.83 ± 3379.44	3394.86 ± 3916.90	1811.44 ± 1034.49	0.930
Serum progesterone (pmol/l)	39.58 ± 21.83	39.90 ± 24.51	38.74 ± 13.71	0.854
Serum LH (IU)	5.02 ± 5.07	5.11 ± 5.89	4.83 ± 2.78	0.291
Dose of FSH stimulation (IU)	1716.13 ± 775.20	1790.91 ± 842.10	1533.33 ± 582.29	0.836
Retrieved oocytes (n)	8.16 ± 4.87	7.23 ± 4.86	10.44 ± 4.30	0.164
Duration of stimulation days	4.03 ± 1.92	3.55 ± 1.50	5.22 ± 2.39	0.266
Matured oocytes (n)	6.94 ± 4.59	6.00 ± 4.51	9.22 ± 4.15	0.406
Viable (Grade I) embryo (n)	4.03 ± 3.41	3.50 ± 3.41	5.33 ± 3.24	0.373
Transferred embryo (n)	1.71 ± 1.35	1.73 ± 1.55	1.67 ± 0.71	0.768
Serum hCG on day 12 (IU)	209.33 ± 442.26	22.97 ± 80.52	664.87 ± 622.48	0.000
Chemical pregnancy n (%)			11(35.5)	
Clinical pregnancy n (%)			9 (29.0)	

*FSH* Follicle-stimulating hormone, *hCG* human Chorionic Gonadotropin, *IVF* In-Vitro Fertilization, *LH* Luteinizing hormone

### Sample collection and preparations

FF and GCs were obtained by follicle puncture at oocyte retrieval. The collected FF was centrifuged for 10 min at 252 x g and the untreated supernatants were frozen and stored at -80 °C until analysis. For GCs FF sediments were incubated in G-IVF™ solution for 2 hours. The mixture was subjected to mechanical and enzymatic treatment in G-Mops™ solution to cleanse the oocytes. At the end of this procedure the sediment contained GC concentrate. Zero point five milliliter of this concentrate was injected into DNA/RNA LoBind Tube and 1 ml ExtraZol Tri-reagent (EM30–200 NucleotestBio Budapest, Hungary) was added. This mixture was incubated in room temperature for 10 min, then stored at-80 °C for future analysis.

### Total RNA isolation and Q-RT-PCR

One hundred microliter of follicle fluid/400 μl of GC suspension was used for RNA isolation. Total cellular RNA was isolated using the ExtraZol Tri-reagent (EM30–200 NucleotestBio Budapest, Hungary) according to the manufacturer’s standard procedures. The primary sequences of the internal control (housekeeping gene) hypoxanthine phosphoribosyltransferase 1 (HPRT 1) were designed with Primer Express™ Software (Applied Biosystemes, Budapest, Hungary) and synthesized by Integrated DNA Technologies (Bio-Sciences, Budapest, Hungary). The primer sequences are shown in Table 2.

**Table 2:** Sequence of primers used for Q-RT-PCR analysis

*Gene name*	*Forward primer sequences (5′-3′)*	*Reverse primer sequence (5′-3′)*
BCL2	GGCCAGGGTCAGAGTTA	CCTCTCTTGCGGAGTATTTG
LHR	GAGATGCACTGTCCAATCC	CCGAATCGAGAGCTGTAATG
FSHR	GGAAGAATCAGGTGGATGG	GGGAGGTCAGAAGGAATCT
AMH	CTTCCTGGAGACGCTCA	GGTCCGACAGGTTGACTA
AMHR2	GGGCTTTGGGCATTACTT	TGTAGAGGCAGCGGATAG
CYP19A1	CAGTGCCTGCAACTACTAC	CCCGAATCGAGAGCTGTAATG
CASP3	CTGAGCCATGGTGAAGAAG	CGGCAGGCCTGAATAATG
CASP8	CCAGTGGGCAAGAGAATTAG	CAAGTGACCAACTCAAGGG
BAX	GAGCTGCAGAGGATGATTG	GCCTTGAGCACCAGTTT
HPRT1	TGCTTCTCCTCAGCTTCA	CTCAGGAGGAGGADGCC

*Abbreviations*: *BCL2* BCL2 apoptosis regulator, *LHR* Luteinizing hormone receptor, *FSHR* Follicle stimulating hormone receptor, *AMH* Anti-Müllerian hormone, *AMHR2* Anti-Müllerian hormone receptor 2, *CYP19A1* Cytochrome P450 family 19 subfamily A member 1, *CASP3* Caspase 3, *CASP8* Caspase 8, *BAX* BCL2 associated X apoptosis regulator, *HPRT1* Hypoxanthine phosphoribosyl transferase 1 (housekeeping gene)

The analysis of gene expression was performed by quantitative RT-PCR using a Roche LightCycler 480 Instrument I (Roche Molecular Systems, Inc. Budapest, Hungary). The thermo-program has been set by the KAPA SYBR® FAST One-Step kit (KK4681, Merck, Hungary) protocol.

The resulting reaction mixture was measured: 10 μl/cell KAPA SYBR FASTqPRC Master Mix, 0.4 μl/cell KAPA RT Mix, 0.4 μl/cell dUTP, 0.4 μl/cell primers, sterile bidest water, 5 μl/cell template mRNA.

The PCR thermocycling conditions were as follows: Reverse transcription step at 42 °C for 5 min follows the enzyme activation 95 °C for 3 s. The PCR reactions were carried out for 40 cycles that comprised a denaturation step at 95 °C for 10 s, an annealing step at 58 °C for 20 s and an extension step at 72 °C for 5 s. The results were analysed by the relative quantification (∆∆_CT_) method [19].

### Statistical analysis

Statistical analysis was performed using IBM SPSS 24.0 software (IBM Corp., Armonk, NY, USA). Normality of data distribution was tested by the Kolmogorov–Smirnov test. To compare continuous variables Mann-Whitney U-test or Wilcoxon W-test were used. The association between two continuous variables was tested by using Spearman’s or Pearson’s correlation coefficients. The data are expressed as mean ± SD and *p* < 0.05 was considered statistically significant.

## Results

**Fig. 1:**
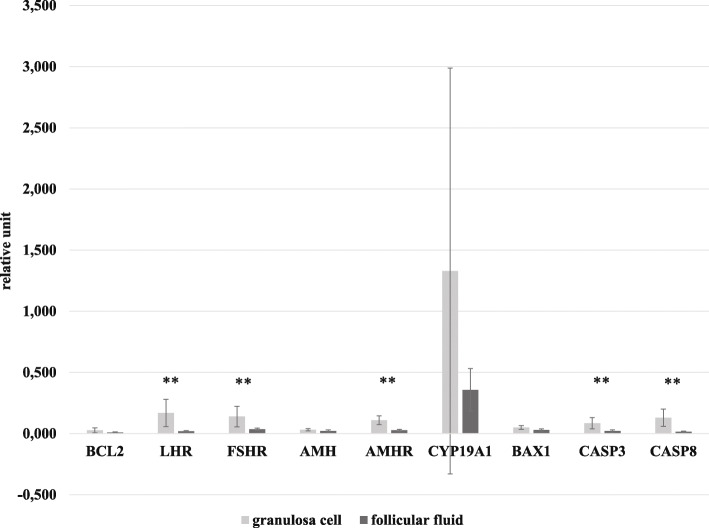
Gene expression levels of anti- and pro-apoptotic factors in granulosa cells and follicular fluid in IVF patients (*n* = 31, mean ± SD). **p ≤ 0.001. Abbreviations as in Table 2

The mRNA expression of anti-apoptotic (BCL2, LHR, FSHR, AMH, AMHR2, CYP19A1) and pro-apoptotic (CASP3, CASP8, BAX) factors in GCs and FF of the whole patient population is shown in Fig. 1. It can be seen that each transcript studied is present in both GCs and FF, but except for BCL2, their FF levels are markedly reduced when compared to those in GCs.

To assess the possible contribution of mRNA expressions measured in GC to their respective levels in FF we analysed the relationship between the corresponding parameters of GC and FF. As we could not demonstrate significant association between transcripts obtained simultaneously from GC and FF our results can be regarded as indicating that these transcripts may originate from several follicular cells including the GC.

Our results were also evaluated according to the IVF outcome (Table 3). Patients with clinical pregnancy (9 patients) were compared with those who failed to become clinically pregnant (22 patients) and we could not detect significant differences between the groups either in GS or in FF expression of pro-or anti-apoptotic factors. However, anti-apoptotic AMH, AMHR2, LHR and FSHR in GC tended to be higher in the pregnant than in the non-pregnant group.

**Table 3:** Comparison of gene expression of anti- and pro-apoptotic factors in pregnancy-positive and pregnancy-negative IVF patients (mean ± SD)

		BCL2		LHR		FSHR		AMH		AMHR		CYP19A1		BAX		CASP3		CASP8	
		GS	FF	GS	FF	GS	FF	GS	FF	GS	FF	GS	FF	GS	FF	GS	FF	GS	FF
Pregnancy- positive	Mean	0.033	0.008	0.113	0.015	0.113	0.033	0.032	0.018	0.147	0.023	0.165	0.566	0.057	0.036	0.089	0.010	0.116	0.018
	SD	0.073	0.010	0.146	0.008	0.114	0.019	0.030	0.016	0.078	0.019	0.169	0.869	0.041	0.029	0.176	0.008	0.172	0.022
Pregnancy- negative	Mean	0.023	0.009	0.191	0.021	0.149	0.037	0.032	0.023	0.093	0.028	1.806	0.278	0.047	0.027	0.082	0.025	0.135	0.014
	SD	0.052	0.014	0.363	0.012	0.276	0.030	0.027	0.026	0.109	0.021	5.560	0.256	0.044	0.021	0.113	0.032	0.214	0.013
	Z	−1.219	−0.221	−0.131	−0.922	−0.261	−0.221	−0.152	0.000	−1.915	−0.516	−1.480	−0.590	− 0.696	−1.012	− 0.044	− 0.516	− 0.305	−0.221
	*p*	0.223	0.825	0.896	0.357	0.794	0.825	0.879	1.000	0.056	0.606	0.139	0.555	0.486	0.312	0.965	0.606	0.761	0.825

*Abbreviations* as in Table 2: *GS* granulosa cell, *FF* follicular fluid

No consistent changes were associated with pregnancy in any other apoptosis-related gene expression investigated either in GC or in FF.

The interrelationship between mRNA expression of pro- and anti-apoptotic factors was also analysed. Interestingly, significant positive relationship was found of GC BAX to GC AMH, AMRH2, LHR and to FSHR. GC CASP3 was also related to AMHR2, to LHR and to FSHR. Similar associations were observed between GC CASP8 and AMHR2, LHR and ESHR (Table 4). These associations apply to the whole IVF population and to the patients of the pregnant group but not to those who did not conceive and progress to clinical pregnancy. Based on these results it is reasonable to assume that apoptotic process initiates increase of gene expression of anti-apoptotic factors to protect cell survival.

**Table 4:** Interrelationship of the mRNA expression of pro- and anti-apoptotic factors in granulosa cell

	AMH	AMHR2	CYP19A1	LHR	FSHR	BCL2
**All patients (*n*** **= 31)**						
BAX						
R	0.360^*^	0.537^**^	−0.062	0.620^**^	0.398^*^	0.672^**^
*p*	**0.046**	**0.002**	0.742	**0.000**	**0.027**	**0.000**
CASP3						
R	0.303	0.509^**^	−0.364^*^	0.692^**^	0.555^**^	0.693^**^
p	0.097	**0.003**	**0.044**	**0.000**	**0.001**	**0.000**
CASP8						
R	0.301	0.585^**^	−0.316	0.655^**^	0.551^**^	0.668^**^
*p*	0.099	**0.001**	0.083	**0.000**	**0.001**	**0.000**
**Pregnancy-positive (*n*** **= 9)**						
BAX						
R	.373	.553^**^	.051	.570^**^	.529^*^	.670^**^
*p*	.087	**.008**	.822	**.006**	**.011**	**.001**
CASP3						
R	.224	.493^*^	−.227	.709^**^	.654^**^	.715^**^
p	.317	**.020**	.311	**.000**	**.001**	**.000**
CASP8						
R	.146	.789^**^	−.226	.828^**^	.773^**^	.805^**^
*p*	.516	**.000**	.312	**.000**	**.000**	**.000**

Abbreviations as in Table 2

^*^p ≤ 0.005

^**^p ≤ 0.001

To get more insight into the clinical relevance of apoptosis in IVF treated patients selected clinical and laboratory variables including parameters of IVF outcome were investigated as a function of apoptosis markers. It was demonstrated that in the whole IVF population maternal age, BMI, FSH dosage for stimulation, number of mature oocytes and hCG levels on day 12 proved to be independent of apoptosis markers. However, the number of retrieved oocytes, was positively related to GC AMHR2 gene expression (*r* = 0.393, *p* = 0.029), whereas the day of embryo transfer was negatively related to the mRNA expression of GC LHR (*r* = − 0.414, *p* = 0.020) and FSHR (*r* = − 0.535, *p* = 0.002). When only patients of the pregnant group were considered significant negative association was found of GC BAX transcript to the number of IVF cycles (*r* = − 0.694, *p* = 0.038) and FF transcripts of CASP3 to number of retrieved (*r* = − 0.841, *p* = 0.036) and mature oocytes (*r* = − 0.833, *p* = 0.020). Furthermore, the number of transferred embryos was negatively affected by mRNA expression of FF BAX (*r* = − 0.920, *p* = 0.008) and FF CASP8 (*r* = − 0.926, *p* = 0.008).

Out of anti-apoptotic factors only GC FSHR and AMHR2 transcripts influenced negatively the day of embryos transfer (*r* = − 0.860, *p* = 0.003) and the number of transferred embryos (*r* = − 0.926, *p* = 0.008), respectively.

## Discussion

The present study demonstrated no significant differences in GS or FF mRNA expression of pro-and anti-apoptotic factors between IVF patients with or without clinical pregnancy. Each pro-apoptotic (BAX, CASP3, CASP8) and anti-apoptotic (BCL2, AMH, AMHR2, CYP19A1, LHR, FSHR) transcripts investigated could be detected in FF, but their levels were markedly reduced and proved to be independent of those in CG. Furthermore, some selected measures of IVF success (number of retrieved and mature oocytes, the day and number of embryo transfer and the number of IVF cycles) were associated with one or more apoptosis-related gene expression. The study, therefore, provided only marginal evidence for the apoptosis dependence of IVF outcome. Importantly, highly significant positive correlations were seen between mRNA expression of pro- and anti-apoptotic factors giving rise to the possibility that the apoptotic process induced adaptive increases of the anti-apoptotic gene expression to attenuate apoptosis and to protect cell survival.

The concept that reduction in apoptotic granulosa-lutein cells in women undergoing IVF treatment is associated with better outcome appears to be well-established. In support of this notion Oosterhuis et al. using flow-cytometry reported 7.1 ± 5.1% apoptotic cells in pregnant as compared to the 20.7 ± 13.7% in non-pregnant patients [1]. Similarly, Nakahara et al. have shown that apoptotic GCs cells estimated by fluorescence microscopy were lower when women conceived, and ongoing pregnancy was achieved [2]. The study by Seifer et al. has also indicated that apoptosis is a measure of ovarian reserve and reflects reproductive potential [20]. Furthermore, evidence has been provided that apoptosis can be associated with poor embryo quality [21]. Comparison of GC apoptosis and clinical outcome of IVF patients with normal and diminished ovarian reserve has also revealed that higher rate of CG apoptosis resulted in a significant reduction in the number of retrieved oocytes and good quality embryos [10]. Consistent with these observations induction of apoptosis of human GCs obtained from IVF patients with interferon gamma and an anti-human Fas antibody showed that low percentage of apoptotic GCs (11.6 ± 4.8% vs 59.5 ± 14.8%) yielded significantly higher pregnancy rate, consequently GC apoptosis might serve as a marker of IVF outcome [22].

In spite of accumulating evidence for the involvement of apoptosis in reproduction its major role in regulating ovarium reserve and IVF outcome has been questioned. Accordingly, GC apoptosis is regarded to be a physiological process to eliminate unwanted cells during the period of folliculogenesis, oogenesis and embryogenesis. In support of this view no significant differences were observed in the percentage of apoptotic GCs and apoptotic cumulus cells between follicles with or without fertilized oocytes [14, 23, 24]. Furthermore, in a series of recent reports by Regan et al. it has been found that the aging- related reduced expression and dysregulation of GC receptors for anti-apoptotic FSHR, LHR and BMPA (bone morphogenetic protein) were associated with lower levels of apoptotic GCs than those in younger women [15, 25, 26].

To reconcile these apparent controversies the contribution of patients’ selection bias, different methodologies and developmental stages, as well as post-translational modifications in the signalling pathways are to be considered. Our present study may shed light to an unexplored possibility. The apoptotic process induces an adaptive increase of the expression of anti-apoptotic genes (AMH, AMHR, FSHR, LHR) that counterbalance the effects of pro-apoptotic factors, re-establish the equilibrium between the mRNA abundance for cell death and survival thus promoting IVF success.

As GCs apoptosis is an integral part of normal development for its progression to reproductive failure interference of some genetic and/or environmental factors are to be assumed. In this regard underlying conditions of infertility, IVF technologies and assisted reproduction-induced epigenetic alterations are to be considered [27–29].

To get better insight into the genetic background of GC apoptosis microarray expression techniques have also been applied to reveal complete genetic profile and to identify genes involved in the apoptosis process. With respect to the bidirectional communication between GCs and oocytes this approach may help to predict the developmental potential of oocytes for successful fertilization and embryo formation [30, 31]. Most recently Chermula et al. using microarray analysis evaluated the gene expression profile of human cumulus cells in long-term in vitro culture. Levels of 133 relevant transcripts were measured on day 1, 7, 15 and 30 of culture. According to the time-course of gene expression three groups were selected; genes in group 1 decreased significantly, in group 2 they increased slightly, while in group 3 there was a marked increase. Six genes of this latter group proved to have an impact on oocyte quality [32].

The presence of apoptosis-related mRNAs in FF needs to be commented on. It was assumed that these transcripts are released from CGs, reflect apoptotic state of GCs and can be used as biomarkers of oocyte maturation, embryo development and pregnancy rate in IVF patients. However, we failed to document such associations suggesting that they derive not only from GCs but also from other follicular cells. Furthermore, except for FF AMH and AMHR, mRNAs in FF have no consistent impact on IVF success. Importantly, cell-free DNA [33, 34] and soluble Fas levels in FF [35] correlated positively with GC apoptosis and negatively with embryo quality and pregnancy rate.

## Conclusions

Taken together, in the light of our present observations we are reluctant to claim decisive role for apoptosis in impaired ovarian function and reproductive potential, but rather we line up with those who challenge its major contribution.

### Study limitations

Relatively small number of patients with heterogeneous infertility diagnosis was included in this study. Moreover, apoptotic GCs were not identified and counted, and the possible post-translational modifications of the gene products were not considered. Further large-scale studies are to be conducted to overcome these practical and theoretical bias.

## Data Availability

The dataset supporting the conclusions of this article is available from the corresponding author on reasonable request.

## Abbreviations

AMH: Anti-Müllerian hormone
AMHR2: Anti-Müllerian hormone receptor 2
BAX: BCL2 associated X apoptosis regulator
BCL2: Apoptosis regulator
BMI: Body Mass Index
CASP3: Caspase 3
CASP8: Caspase 8
CYP19A1: Cytochrome P450 family 19 subfamily A member 1
FF: Follicular fluid
FSH: Follicle-stimulating hormone
FSHR: Follicle stimulating hormone receptor
GCs: Granulosa cells
hCG: Human chorionic gonadotropin
HPRT1: Hypoxanthine phosphoribosyl transferase 1 (housekeeping gene)
IVF: In vitro fertilization
LHR: Luteinizing hormone receptor
*Q-RT-PCR*: Real-Time Quantitative Reverse Transcription Polymerase Chain Reaction
